# Unraveling the Particle Morphology of ABS Polymer Latexes by 3D STEM

**DOI:** 10.1021/acs.macromol.5c00657

**Published:** 2025-08-06

**Authors:** Ainara Agirre, Evgeny Modin, Andrey Chuvilin, Miren Aguirre, Jose R. Leiza

**Affiliations:** 1 POLYMAT eta Kimika Aplikatua saila, Kimika Fakultatea, University of the Basque Country (UPV/EHU), Joxe Mari Korta Zentroa, Tolosa Hiribidea 72, Donostia-San Sebastián 20018, Spain; 2 CIC nanoGUNE BRTA, Tolosa Hiribidea 76, Donostia-San Sebastián 20018, Spain; 3 IKERBASQUE, Basque Foundation for Science, Plaza Euskadi 5, Bilbao 48009, Spain

## Abstract

Acrylonitrile–butadiene–styrene (ABS) materials have a complex microstructure formed by a dispersion of grafted polybutadiene (PB) particles in a poly­(styrene-acrylonitrile) matrix (SAN). The grafting properties of the grafted PB particles are critical to achieving effective dispersion and compatibility with the SAN matrix in the compounded ABS material. Therefore, characterization of the particle morphology helps understand the polymerization mechanism, as well as the final properties of the ABS material. In this work, we utilize high angle annular dark-field scanning transmission microscopy (HAADF-STEM) tomography to determine the morphology of ABS polymer latex particles. Electron tomography (ET) 3D reconstruction of the whole particle allows determining quantitatively the volume fractions and distribution of each phase (internal SAN clusters, PB matrix, and SAN shell) in the latex particle. The information acquired in a single particle is used to illustrate that internal and external grafting properties can be determined accurately from microscopic analysis. The total grafting (internal plus external) agrees with the grafting properties measured experimentally by an extraction process.

## Graphical abstract

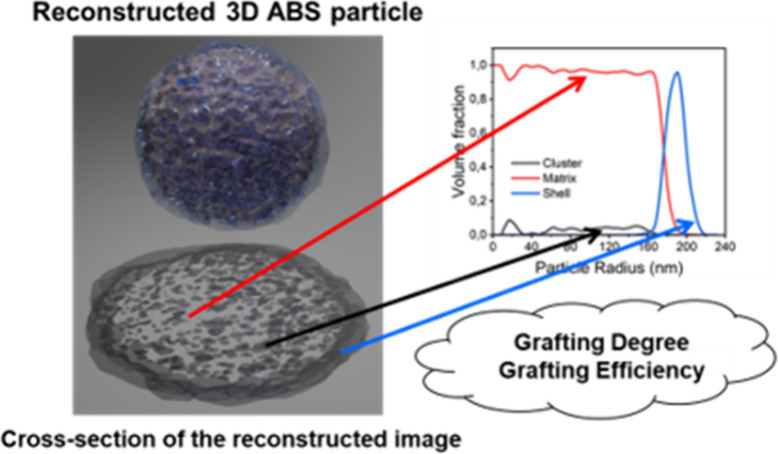

## Introduction

ABS is an engineering plastic that is produced by compounding a poly­(styrene-*co*-acrylonitrile) copolymer (SAN) that acts as the matrix where SAN-grafted polybutadiene (PB) particles (grafted-ABS) produced by bulk or emulsion polymerization processes are dispersed. Thus, the ABS material is elastomeric and thermoplastic, and its compositional flexibility offered by the use of a three-monomer system, combined with the option to alter structural and compositional parameters during the synthesis of the polymer, allows the polymer to be customized to meet the specific requirements of the product.^1–4^

Figure 1 displays the microstructure of the ABS material. During the grafting reaction, which can be carried out by an emulsion polymerization process, SAN chains (light blue) are grafted onto the PB backbone chains (orange), but free SAN chains (dark blue) are also formed. The SAN chains can be grafted, forming occlusions or clusters (internal grafting), which are dispersed in the PB matrix, or can be grafted around the PB matrix (external grafting), which along with the free SAN chains form the shell surrounding the particle.^5–7^ In this way, ABS polymer particles with a morphology similar to the one schematically shown in the inset of Figure 1 are obtained.

**Figure 1:**
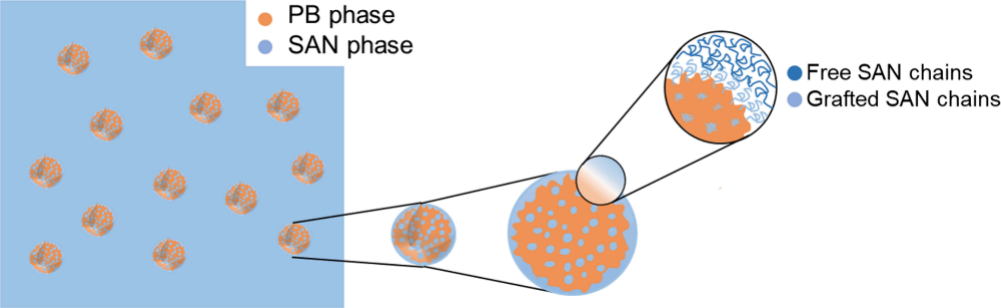
Multiphase structure of the ABS compound polymer containing grafted PB particles dispersed in a SAN matrix.

As mentioned, the grafting of the SAN onto the PB is critical to achieving an effective dispersion and compatibility between both phases in the final compounded ABS material. Therefore, the grafting will define not only the final particle morphology but also the properties of the material, and hence, the characterization of the microstructure in terms of particle morphology is of paramount importance.

A great variety of techniques have been used to characterize the particle morphology, but electron microscopy (EM) is by far the most used, versatile, and powerful instrument for the complete microstructural analysis of polymers.^8,9^ In the literature, different EM techniques have been used, and each one provides different information regarding the microstructure of the polymer as it will be summarized in the following lines.

First, it is worth mentioning that at the advent of EM, the characterization of the fine microstructure of biphase particles, which contained a soft phase, such as PB rubber, was challenging for two reasons: first, the preparation of ultrathin sections of soft polymers was elusive because the sample was usually deformed during the preparation process, and second, the difficulties in obtaining high-contrast images in which the two polymer phases could be clearly distinguished. However, in 1965, Kato developed for the first time a method that overcame these difficulties and allowed the characterization of these soft microstructures while avoiding the destruction of their original microstructure.^10^ The method consisted of staining the polymer with osmium tetroxide (OsO_4_) vapor, which resulted in an excellent fixation and staining effect of the PB double bonds, since the OsO_4_ reacts selectively with the double bonds of the unsaturated polymers. This allowed the characterization of the particle size, particle size distribution, and microstructure without the need for additional techniques. In addition, the study of the internal morphology of the compounded ABS materials had several difficulties, such as uncontrollable structural changes, susceptibility to electron beam damage, difficulties to be sectioned, and poor image contrast.^11^ However, Kato went further characterizing the internal morphology of the compounded ABS materials by preparing ABS blocks, which were obtained from the processing of grafted-ABS together with a SAN matrix, staining with a diluted OsO_4_ vapor, and later sectioning without any embedding procedures. Likewise, Kato published several articles on the internal characterization of compounded ABS polymers using the method described above.^11–14^

In the following years, several works were carried out on the characterization of ABS polymers by EM, and most of them followed Kato’s method during the preparation of the sample.^13–22^ Most commonly, particle size, particle size distribution, colloidal dispersion, and particle morphology were analyzed. Transmission electron microscopy (TEM) has been by far the most widely used technique; however, scanning electron microscopy (SEM)^16,24,25^ has also been used over the years for the characterization of the ABS microstructure, among others.

The morphology of the ABS polymer is usually based on a core–shell microstructure, and this has been corroborated by many authors since the 1970s.^1,5,7,16,18,20,23,25–27^ As it has been mentioned, the ABS material is composed by a multiphase microstructure that enables the compatibility between both SAN and PB phases. What mainly prevents the PB particles from aggregation due to the van der Waals attraction forces is the fact that the surface of these particles is completely covered by a layer of SAN. Therefore, a minimum thickness of the grafted SAN in the shell is necessary to ensure a good dispersion of the polymer particles in the final compounded material, and the greater the thickness of the grafted SAN layer, the better is the dispersion of these particles in the final ABS material.^1,26,28^

In this vein, Bertin et al.^26^ studied the relationship between the viscoelastic properties (storage and loss modulus) and the degree of grafting of ABS materials prepared by blending several SAN polymers as a matrix (different S/AN ratios and molar masses) with SAN grafted PB particles (with different PB particle sizes). The storage modulus presented a minimum with the grafting degree that the authors considered as the critical degree of grafting (GDc). They hypothesized that the degree of grafting affected the morphology of the grafted SAN chains on the surface of the PB particle, assuming that all the grafted material was on the surface. Based on TEM images of the ABS material taken before and after shearing, they concluded that undergrafted (GD < GDc) ABS samples presented a mushroom morphology because the graft ABS could not cover the whole surface area and graft chains did not overlap each other. On the contrary, overgrafted ABS (GD > GDc) presented a brush morphology because the density of the graft point was above the critical value and the graft chains were stretched, and the free chains of the matrix were expelled from the graft layer. In conclusion, the graft morphology, the amount of the grafted SAN per particle, and the formation of a minimum layer of SAN around the PB particle are critical parameters to obtain a good dispersion of the grafted ABS particles in the SAN matrix, which at the same time is of great importance to achieve good properties of the final ABS material.

Moreover, most of the analyses regarding the morphology of the ABS particles reported in the literature were carried out in compounded samples, for example, grafted ABS particles embedded in a SAN matrix. Nevertheless, the morphology of a stand-alone grafted ABS polymer particle and the internal morphology of grafted ABS polymer latex particles have not been reported yet. The main limitation of conventional TEM and SEM imaging techniques is the generation of two-dimensional (2D) data for an object that is actually three-dimensional (3D), which creates an ambiguity in the interpretation and statistical analysis. ET is broadly used in biological and material sciences to characterize the 3D structure and composition of a variety of nanomaterials,^9,15^ particularly, organic–inorganic polymer particles^29,30^ and organic–organic composites.^31^ It is a technique that provides nanometer scale resolution in 3D. ET reconstructs the 3D interior structure of the nanomaterials from a tilt series of 2D projections combined with the necessary computational tools to obtain the 3D image.^8,32^ Mainly, two different nanoscale imaging techniques have been used, TEM and scanning transmission microscopy (STEM), where different illumination modes are utilized, which results in very different contrast mechanisms. Even though both techniques can be used for electron tomography, HAADF-STEM found a broader application for ET^8,9,15,29,31,33,34^ due to its simple monotonic contrast, which simplifies processing of tomograms.

In this work, HAADF-STEM ET, which has been recently implemented to analyze the 3D microstructures of soft polymer latex particles,^31,35^ is used to characterize the internal particle morphology of singular ABS polymer latex particles. The morphology of the particle allowed accurate determination of the volume fractions of each phase and its radial distribution. Additionally, this information has been used to illustrate how the internal grafting degree and grafting efficiency can be directly calculated. Furthermore, using the surface area of the PB matrix and assuming that the thickness of the shell that would be grafted to the PB particles can be related to the radius of gyration of the SAN chains, the external grafting properties can be determined. In other words, the method presented here will allow, if enough particles are analyzed, determining microscopically the total grafting properties (i.e., internal and external grafting) of ABS latex particles.

## Experimental Section

### ABS Latex and TEM Sample Preparation

The grafted ABS latex was synthesized by seeded semibatch emulsion polymerization using a water-soluble initiator. The seed was a PB latex, and styrene and acrylonitrile were added as a pre-emulsion. A drop of ABS latex sample was deposited in a carbon coated standard Cu TEM grid and dried at 5 °C in a fridge. Then, the dried grid was stained using OsO_4_ vapor for 1 h. Note that OsO_4_ reacts only with PB double bonds, and hence, only PB rich phases will contain Os, which, due to its high atomic number, will be seen bright in HAADF STEM images.

### Particle Morphology

A detailed description of the microscopy technique and the steps followed to obtain the 3D reconstruction of the ABS polymer particle were described elsewhere.^31^ Below is a brief summary of the methodology.

A Titan 60–300 electron microscope (FEI Company, Netherlands) operating at an acceleration voltage of 300 kV was used for acquisition of HAADF-STEM tomography data. HAADF-STEM imaging mode provides the contrast that is strongly dependent on the atomic number (∼*Z* ^2^), and thus, the stained polymer phase looks much brighter at HAADF-STEM images. Tilt series were automatically acquired with FEI Tomography 4.0 software at angles between −70° and +70° at a 2° tilt step. The tilt-series alignment and tomographic reconstructions were performed using in-house DigitalMicrograph (Gatan) scripts. For the stained phase separation, the intensity-based segmentation (local thresholding criteria) was used,^31^ which works based on the intensity difference of pixels, and therefore, they were assumed to belong to the stained PB phase (bright) or to the nonstained SAN phase (dark). Therefore, the classification of internal SAN and SAN in the shell (external) is done on the basis of their location (i.e., radial distribution). Furthermore, it is worth pointing out that grafted and free SAN chains cannot be distinguished by this analysis. The segmentation of the different phases present in the particle, the subsequent 3D rendering, and also the statistical calculations were done using Thermo Scientific TM Amira Avizo 3D software. In the Supporting Information, primary and intermediate data (Figure S1) of the tomographic analysis that include a video of the tilt series and snapshots taken at different angles and a video (Video S1) of the reconstructed 3D volume are included.

### Study of the Radial Distribution of Different Phases in the Polymer Particles

A representative illustration of a typical grafted ABS particle synthesized by using water-soluble initiators is shown in Figure 2. More specifically, the ABS particle morphology is composed by a PB core (orange color), in which SAN clusters are distributed (blue color), and a shell composed by SAN lobes or clusters, which partially penetrate into the core (also blue color). In this schematic, a radial distribution of the components plays an important role, as it reflects the peculiarities of the copolymerization dynamics, which allows a better understanding of the formation of this morphology in the grafted ABS polymer particles synthesized by emulsion polymerization.

**Figure 2:**
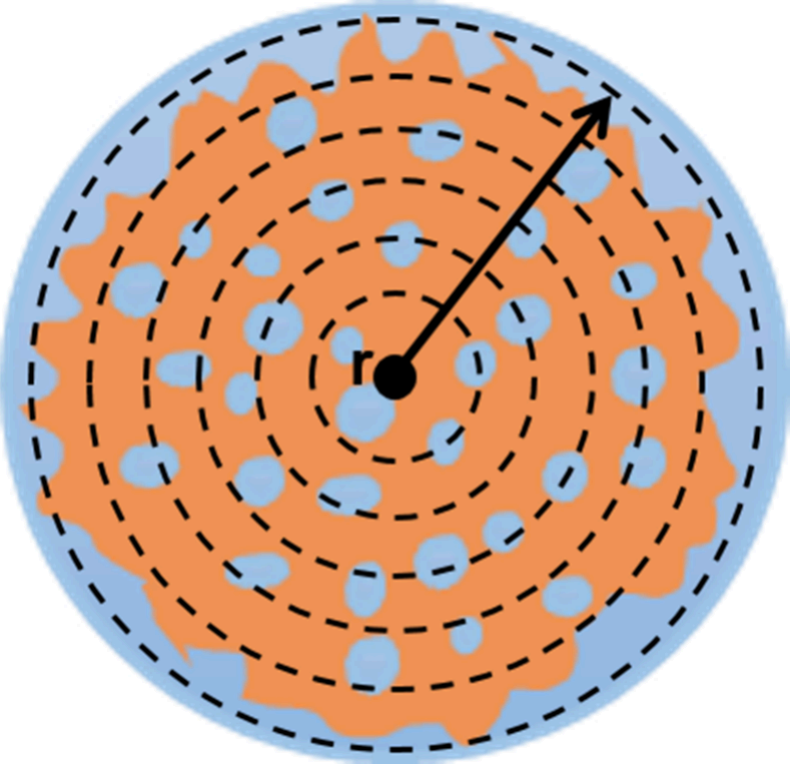
Representative illustration of the radial distribution of an ABS polymer particle.

For that purpose, the following workflow was implemented. At the first step, the 3D data set (single particle tomogram) was segmented and the 3D distribution maps for the three components (internal SAN clusters, PB matrix, and SAN shell) were obtained. 3D phase distribution maps are 3D data sets *I*(*x*,*y*,*z*), where *I* takes the value 1 if the (*x*,*y*,*z*) vortex contains a corresponding polymer phase and 0 in the other case. The center of the particle was calculated as a common center of mass for the three data sets. From this center, the sphere of radius *r* was determined, and the average value of *I*(*x*,*y*,*z*) on this sphere as a function of *r* was calculated for all distribution maps. As determined, function VF­(*r*) corresponds to the average volume fraction of the corresponding phase at distance *r* from the center.

Cluster size analysis (Figure 4) was performed on the binarized distribution map of clusters. Individual clusters were detected, and their positions and volumes were assembled in a data set. The center of latex particle was calculated in this case as a mean value of cluster positions. Based on this data, a distribution of cluster sizes vs radius (distance from the center) was calculated.

### Microscopic Characterization of Internal Grafting Properties

As mentioned in the Introduction, the SAN copolymer chains can be grafted at the surface of the PB particle (i.e., in the shell) or within the PB particle forming aggregates or clusters, which leads to what is known as external or internal grafting, respectively. As noted above, the tomographic analysis does not allow differentiation between free and grafted SAN (both are seen dark); therefore, in both SAN phases, grafted and free SAN chains could be present. Nonetheless, it is likely more probable that the free SAN is present in the shell than in the internal clusters.

If it is assumed that occlusions or clusters are internally grafted SAN chains and that the externally grafted and free SAN chains compose the shell, the characterization of the internal grafting properties can be defined as follows:

a) Internal grafting degree (GD_int_): weight of internally grafted SAN copolymer chains (clusters) with respect to the total weight of the PB core. The weight of each phase was obtained by multiplying the volume of the phase with the corresponding density (ρ_SAN_ = 1.07 g/cm^3^,^36,37^ ρ_PB_ = 0.90 g/cm^3^ ^38,39^ ), as shown in eq 1:
$$\mathrm{GDint}=\frac{V(\mathrm{SAN}\;\mathrm{clusters})\cdot \rho (\mathrm{SAN})}{V(\mathrm{PB}\;\mathrm{phase})\cdot \rho (\mathrm{PB})}$$ 1
b) Internal grafting efficiency (GE_int_): weight of internally grafted SAN copolymer chains (clusters) with respect to the total weight of SAN copolymer (grafted and free) present in the sample. The following equation was used:
$$\mathrm{GEint}=\frac{V(\mathrm{SAN}\;\mathrm{clusters})}{\left[V(\mathrm{SAN}\;\mathrm{clusters})+V(\mathrm{SAN}\;\mathrm{shell})\right]}$$ 2

Therefore, the internal grafting properties were determined using the volumes of SAN clusters, the SAN shell, and the PB core phases obtained by the segmentation of each phase after the electron tomography analysis.

## Results and Discussion

### Characterization of the Particle Morphology

Figure 3 presents a 2D HAADF-STEM image of the ABS particle and a slice of the reconstructed image where the internal morphology of the ABS particle can be better visualized than that in the 2D image. In the Supporting Information (Figures S2 and S3), overview 2D images at lower magnification are also provided for visualization of a larger number of ABS particles. Additionally, the 3D reconstructed model of the particle and 3D models and cross sections of distribution maps of the inner SAN clusters, PB matrix, and external SAN shell are shown.

**Figure 3:**
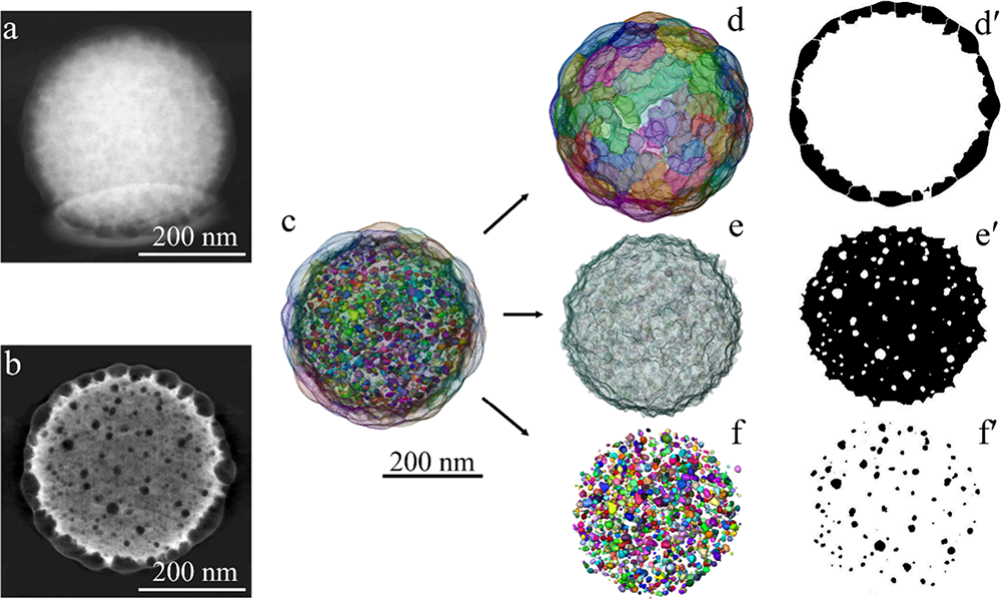
(a) 2D HAADF-STEM micrograph of the ABS dry polymer latex particle. (b) Slice of the 3D tomographic reconstructed ABS polymer particle. (c) Reconstructed 3D model: (d, d′) SAN shell and section of the SAN shell; (e, e′) PB core and section of the PB core; and (f, f′) internal SAN clusters and section with the internal SAN clusters.

Note that in HAADF-STEM, the polybutadiene rich phase appears brighter than the SAN rich phase as explained earlier. From the slice of the reconstructed image, it can be clearly seen that the equilibrium “core–shell” morphology was not attained, since the SAN copolymer is distributed between the shell surrounding the PB core and small nano domains (clusters) within the PB particle, which were not moved to the equilibrium position (surface of the particle) due to the grafting of the internal SAN chains onto the PB core. There are a large number of internal clusters of SAN of irregular shape and sizes of few nanometers (see details below) present in the PB core. In addition, it can be seen that the SAN clusters on the surface maintain their identity and do not form a uniform round shell, presenting a protrusion of SAN lobes that did not coalesce during the reaction. It should also be taken into account that the SAN clusters on the surface of the particle seem to partially penetrate into the matrix as the PB matrix is soft. This partial diffusion of the hard SAN clusters causes deformation of the PB core. Similar results were obtained by Rajabalinia et al.^31^ for a multilobed composite latex particle composed of an acrylic core and styrene-acrylate phases synthesized by seeded semibatch emulsion polymerization.

The detailed particle morphology in Figure 3 was further analyzed by 3D statistics. Before analyzing this detailed information, a comparison of the composition calculated from the microscopic data and the one corresponding to the formulation used in the emulsion polymerization was done. Table 1 shows the weight fractions of SAN (internal clusters plus shell) and PB calculated from the segmentation statistics and the values of the formulation. The weight fractions displayed in Table 1 for the tomographic analysis were obtained from the volume fractions and corresponding phase densities.

**Table 1:** Comparison between the SAN and PB Weight Fractions of the ABS Polymer Particle Calculated from the Results Obtained by HAADF-STEM Tomography and from the Formulation Used to Synthesize the Latex

	tomography^a^		formulation	
	**SAN (%)**	**PB (%)**	**SAN (%)**	**PB (%)**
ABS polymer particle	38.6	61.4	39	61

a The volumes (in μm^3^) of each phase are *V*_PB, matrix_ = 2.1 ×10^–2^, *V*_SAN, shell_ = 9.9 ×10^–3^, and *V*_SAN, clusters_ = 1.3 *×* 10^–3^.

Both values agree, demonstrating that the ET technique based on Os staining and HAADF STEM imaging gives unbiased estimation of bicomponent particle composition. However, larger statistics (i.e., larger number of polymer particles analyzed) will be needed to confirm this agreement. It is also worth noting that in perfectly mixed stirred tank reactors such as those used to synthesize these particles, the particle properties are homogeneous.

Statistics of the segmentation analysis provide interesting information about the morphology of the particle and its formation. Figure 4a shows the radial distribution of the volume fraction of three distinctive phases present in the particle, namely, the PB core phase, the SAN internal clusters, and the SAN in the shell. The PB core (matrix, red line) is present from the center of the particle (0 nm) up to a radial distance of around 190 nm with high volume fractions. On the other hand, the presence of clusters, which is represented with the black line, starts from the center up to 170 nm. Finally, the shell is presented from a radial distance between 160 and 220 nm. These distributions shed light on the profile of radical concentration along the particle radius and the loci of the polymerization of the SAN copolymer during the semibatch polymerization. The fact that the sum of the internal and shell SAN will have a very sharp shape from the surface of the particle toward the center indicates that radicals entering from the aqueous phase are anchored in the surface and most of the SAN polymer is formed in the surface of the particle creating large lobes. Nonetheless, some radicals diffuse inward where they grow and are grafted in the PB chains.

**Figure 4:**
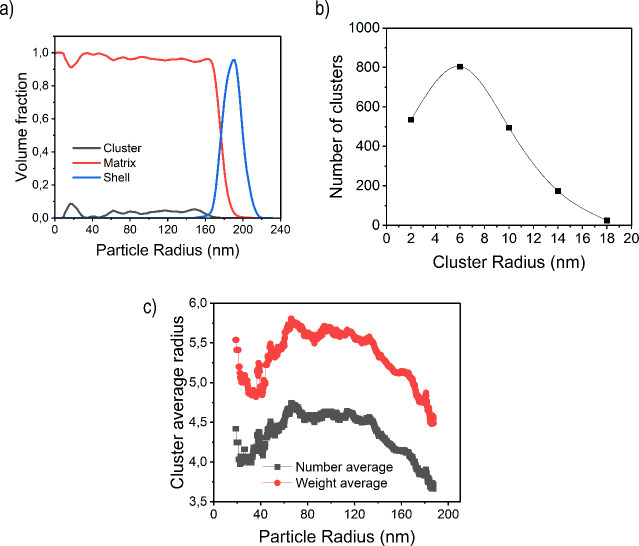
(a) Volume fraction of each polymeric phase against the radius of the ABS polymer latex particle obtained from the segmentation of the tomographic reconstruction; (b) number distribution of the cluster sizes in the ABS polymer particle; (c) internal cluster number and weight-average radius as a function of the radial distance of the particle.

Figure 4b presents the size distributions of the internal clusters. The radius of the clusters is small (between 2 and 18 nm), which indicates that little aggregation of clusters occurs in the particle because most of them are covalently grafted and, hence, their diffusion is hindered.

Finally, Figure 4c shows the number and weight-average radius of the internal SAN cluster as a function of the particle radius. For a more detailed radial distribution of cluster sizes, see the Supporting Information (Figure S3). According to this radial distribution, the larger clusters are located at 100–140 nm from the surface of the PB seed particles. So far, mathematical models that predict the evolution of particle morphology of seeded semibatch emulsion polymerization including the radial distribution of internal domains are scarce,^40^ but they do not consider phase separation and evolution of the domains. Other models available in the literature^31,41^ consider phase separation and coagulation of the clusters and, hence, their dynamics and size distribution, but unfortunately not their radial distribution. Therefore, the tomographic information, illustrated in this work for this particular ABS particle, will allow us to validate future upgraded models that considered all the features affecting the morphology development mentioned above. Thus, uncertainties about the penetration of the oligoradicals into the particle and the likelihood of internal cluster formation (by grafting and phase separation) and aggregation will benefit from the detailed and unique information gathered by tomography.

In addition, Figure 4a shows that if the volume fraction of each phase is analyzed, it can be observed that the fraction of clusters (the amount of SAN copolymer grown and grafted in the interior of the PB core) is low because of the limited diffusion of the growing radicals from the surface to the interior of the particle. Interestingly, it can be observed that the SAN shell and the PB core overlap substantially in the outer part of the particle (between 170 and 190 nm), which suggests that most of the SAN has grown in the surface of the polybutadiene rather than within the particle.

### Microscopic Characterization of the Internal and External Grafting Properties

The information gathered from the 3D statistics can be used directly to obtain information about the internal degree of grafting and grafting efficiency. Herein, Table 2 presents the internal grafting values (calculated from eqs 1 and 2).

**Table 2:** Internal GD and GE Values Obtained for the Polymer Particle Analyzed by HAADF-STEM Tomography

	**GD**_**int**_ **(%)**	**GE**_**int**_ **(%)**
ABS polymer particle	7.3	13.3

The internal grafting values are substantially lower than the total macroscopic grafting values reported in the literature,^4,42^ which are above 45%. Thus, the internally grafted SAN with respect to the amount of PB is around 7% (i.e., grafting degree) and with respect to the total SAN is around 13% (i.e., grafting efficiency). Therefore, it can be concluded that there are many more SAN chains grafted around the PB core (external grafting) than the SAN chains grafted in the form of clusters (internal grafting), likely because during polymerization there is a sharp profile of radical concentration in the polymer particle (note that a water-soluble initiator was used in the synthesis of the latex), and SAN monomers reacted nearby the particle’s surface, forming lobes that partially penetrate in the soft cross-linked PB core. This explains the overlap of the PB matrix and SAN shell seen in Figure 4a.

A fraction of the SAN, which is in direct contact with the PB matrix surface, is grafted (in light blue color in Figure 5). The volume of this grafted fraction can be estimated in a good approximation as the surface of the PB matrix multiplied by the thickness (ξ_graft–SAN_). The surface of the matrix, which is very irregular, can be directly measured from the segmented 3D distribution map of the PB matrix. This surface was measured as *S*_PB_ = 0.51 μm^2^, which is 33% greater than the area corresponding to a sphere of the original PB seed particle size due to the deformation induced by the SAN lobes in the soft PB core. The thickness of the grafted layer can be assumed to be close to the size of individual SAN chains (or the radius of gyration of the chains^26^), which in turn depends on the degree of polymerization and can be independently estimated by multiangle light scattering. The *z*-average radius of gyration of the soluble SAN chains of the ABS latex is 15.7 nm (see the Supporting Information, Table S1, for details of the characterization) analyzed in an AF4/MALS/RI equipment (asymmetric-flow field flow fractionation coupled with multiangle light scattering and refractive index detectors). Furthermore, the size of the internal clusters of SAN is related with the average degree of polymerization achieved by the radicals that diffuse inward and terminate by grafting in the PB chains. Overall, the kinetic chain length distribution of these internal chains and those created in the shell should be similar. The size distribution of the internal clusters calculated from the segmentation analysis of the TEM reconstructed particle is plotted in Figure 4b. The *z*-average of the cluster radius distribution is 10.6 nm (Supporting Information). The *z*-average radius of gyration obtained from AF4/MALS/SI overestimates the size of the chains, likely because of the cutoff membrane pore size (10 kDa) used in the separation channel of the AF4 equipment, which filters out the smaller chains.

**Figure 5:**
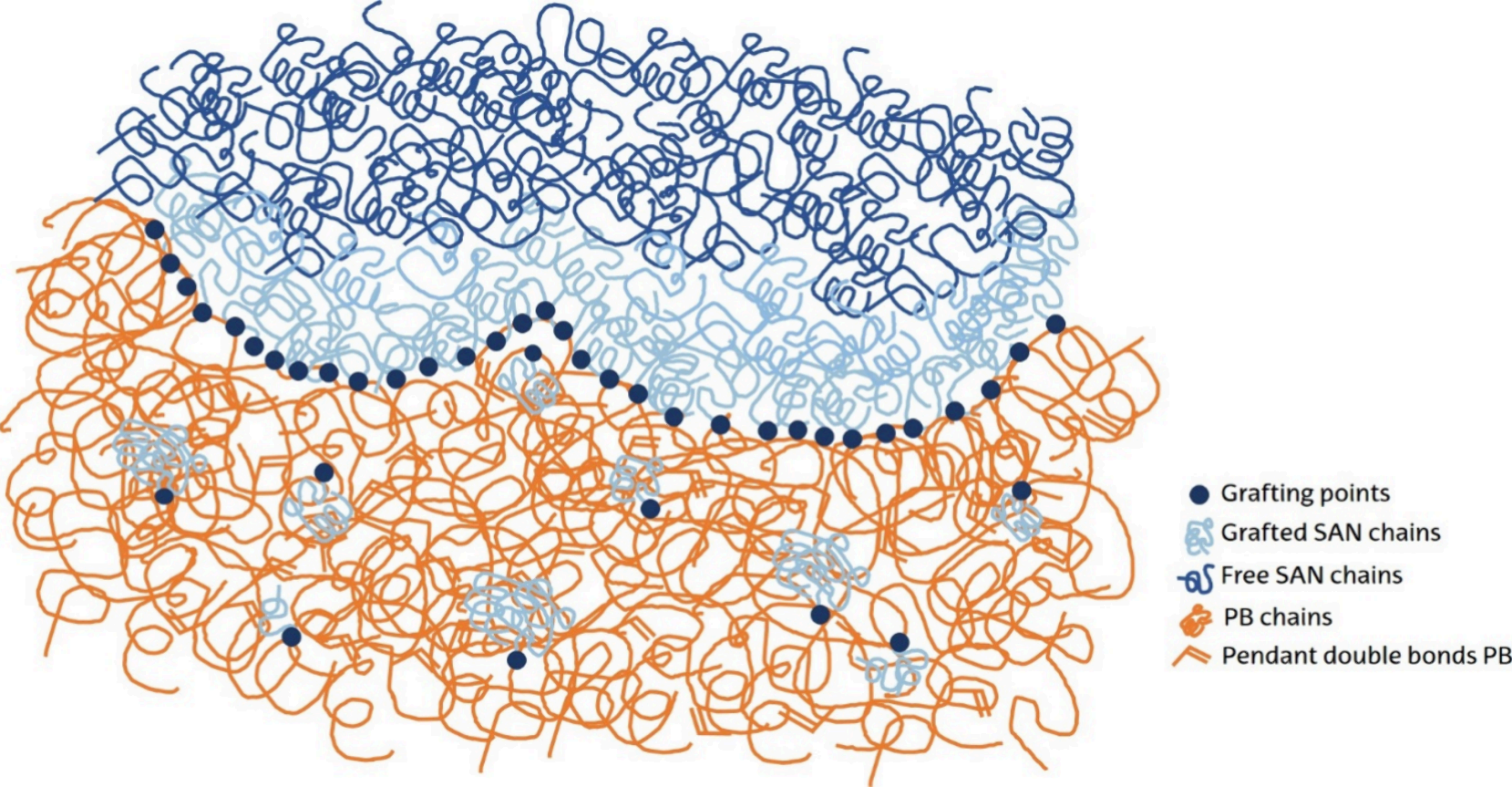
Schematic of the interface of the PB core and the SAN lobe showing a microscopic region (not in scale) of the PB chains, the grafted SAN chains in the PB matrix, and the free SAN chains.

Thus, if the *z*-average radius of the internal clusters is considered as the thickness of the grafted SAN chains, the external grafting efficiency can be calculated microscopically as

$$GE_{ext}=\frac{S_{PB}\;\xi_{graft-SAN}}{V_{SAN-total}}$$ 3

where ξ_graft–SAN_ is the thickness of the grafted SAN chains (nm), *V*_SAN–total_ = *V*_SAN_cluster__ + *V*_SAN_shell__ (μm^3^) (where *V*_SAN_cluster__ and *V*_SAN_shell__ correspond to the volume of the SAN phases in the cluster and in the shell, respectively, as assigned during the segmentation analysis (see Table 1)), and *S*_PB_ is the surface of the PB matrix (μm^2^), also obtained from the segmentation analysis. Using the *z*-average radius of the clusters calculated from the segmentation, a value of GE_ext_ of 48.4% is obtained. If the GE_int_ and GE_ext_ are added, the total grafting efficiency is 62.5%, which is in very good agreement with the overall experimentally grafting efficiency calculated for the ABS latex used in this work, which was 67.4%.^42^

Microscopic analysis of the internal and external grafting properties will allow us to understand the effect of reaction conditions in grafting properties of ABS latexes in a detail that is not possible from the experimental values obtained currently by extraction/centrifugation processes. Also, this detailed microscopic analysis will help improve the knowledge between the microstructure/morphology of the ABS latex particles with the performance of the ABS compounded materials.

## Conclusions

A method for the precise quantitative 3D characterization of the morphology of a grafted ABS polymer particle has been developed. The method was previously proved for soft polymers^29–31^ and is based on HAADF-STEM combined with image reconstruction. The morphology calculated from the reconstructed image of the particle is composed by a PB matrix core where internally grafted SAN chains associated with forming clusters and SAN chains in the surface of the PB matrix in the form of lobes that partially penetrate in the soft PB matrix and deform it. The statistical analysis of the 3D reconstructed particle allows the calculation of the internal size distribution of the clusters as well as the radial distribution of the three distinct phases: the internal SAN, the PB matrix, and the SAN in the shell.

From this information, it has been illustrated how the internal grafting degree and efficiencies can be directly determined, which for the ABS latex analyzed yield values between 7 and 13%, respectively. These values are lower than the typical total grafted properties calculated macroscopically from the extraction of soluble and insoluble fractions of the ABS polymer. In addition, we also show that the external grafting properties can be obtained if the thickness of the layer of SAN chains on the surface of the PB matrix is known. We show that considering the *z*-average radius of gyration of the internal clusters of SAN as this thickness (which is a good approximation of the degree of polymerization of the SAN chains), the total grafting efficiencies calculated microscopically (internal plus external) are in very good agreement with those obtained from experimental values.

In summary, we have shown that 3D STEM is a powerful technique to characterize bicomponent latex particles. We think that by extension of this type of tomographic analysis to samples taken during the polymerization and final samples of processes carried out under different experimental conditions (e.g., different PB particle sizes or different chain transfer concentrations), the information gathered will pave the way for a better understanding of the ABS emulsion copolymerization process, fine-tuning the kinetic and structural models and, as a final goal, optimization of composite properties.

## Supplementary Material





## Notes

Primary and intermediate data of the tomographic analysis including snapshots and low magnification STEM-HAADF and STEM-HAABF images of the ABS latex sample; statistical information on the cluster size distributions in the particle; SEC and AF4/MALS/RI analysis of the soluble fraction of the ABS latex; *z*-average radius of the internal clusters of SAN (PDF)
A video of the tilt series and 3D reconstruction of the particle (MP4)

## References

[1] Bucknall, C. B. Toughened Plastics, 1st ed.; Springer, 1977.

[2] Maul, J. ; Frushour, B. G. ; Kontoff, J. R. ; Eichenauer, H. ; Ott, K.-H. ; Schade, C. Polystyrene and Styrene Copolymers. In Ullmann’s Encyclopedia of Industrial Chemistry; Wiley-VCH Verlag GmbH & Co., 2000.

[3] Adams, M. E. ; Buckley, D. J. ; Colborn, R. E. ; England, W. P. ; Schissel, D. N. Acrylonitrile-Butadiene-Styrene Polymers; Rapra Technology LTD, 1993.

[4] Kulich, D. M. ; Gaggar, S. K. ; Lowry, V. ; Stepien, R. Acrylonitrile-Butadiene-Styrene Polymers. In Encyclopedia Of Polymer Science and Technology; Wiley, 2001; Vol. 1, pp 174–203.

[5] Xu X. F., Wang R., Tan Z. Y., Yang H. D., Zhang M. Y., Zhang H. X. (2005). Effects of Polybutadiene-g-SAN
Impact Modifiers on
the Morphology and Mechanical Behaviors of ABS Blends. Eur. Polym. J..

[6] Sun S., Tan Z., Zhou C., Zhang M., Zhang H. (2007). Effect of ABS Grafting
Degree and Compatibilization on the Properties of PBT/ABS Blends. Polym. Compos..

[7] Zhang N., Bao X. X., Tan Z. Y., Sun S. L., Zhou C., Yang H. D., Zhang H. X. (2007). Morphology
and Mechanical Properties
of ABS Blends Prepared from Emulsion-Polymerized PB-g-SAN Impact Modifier
with AIBN as Initiator. J. Appl. Polym. Sci..

[8] Midgley P. A., Weyland M., Thomas M., Johnson B. F. G. (2001). Z-Contrast Tomography:
A Technique in Three-Dimensional Nanostructural Analysis Based on
Rutherford Scattering. Chem. Commun..

[9] Kübel C., Voigt A., Schoenmakers R., Otten M., Su D., Lee T., Carlsson A., Bradley J. (2005). Recent Advances in Electron Tomography:
TEM and HAADF-STEM Tomography for Materials Science and Semiconductor
Applications. Microsc. Microanal..

[10] Kato K. (1965). Osmium Tetroxide
Fixation of Rubber Latices. J. Electron Microsc..

[11] Kato K. (1965). Electron Microscopy
of ABS Plastics. J. Electron Microsc..

[12] Kato K. (1967). ABS Mouldings
for Electroplating-An Electron Microscope Study. Polymer..

[13] Kato K. (1968). Electron Microscope
Studies on the Etching of ABS Mouldings for Electroplating. Polymer..

[14] Kato K. (1968). Molding Anisotropy
in ABS Polymers as Revealed by Electron Microscopy. Polymer (Guildf)..

[15] Zhong Z., Goris B., Schoenmakers R., Bals S., Batenburg K. J. (2017). A Bimodal
Tomographic Reconstruction Technique Combining EDS-STEM and HAADF-STEM. Ultramicroscopy..

[16] Huguet M. G., Paxton T. R. (1971). ABS Polymer as a
Colloidal Dispersion. Colloidal and Morphological
Behavior of Block and Graft Copolymers.

[17] Bernal C. R., Frontini P. M., Sforza M., Bibbó M. A. (1995). Microstructure,
Deformation, and Fracture Behavior of Commercial ABS Resins. J. Appl. Polym. Sci..

[18] Yamane H., Maekawa Z., Sakano H. (2000). Effect of Rubber Particle
Size and
Graft Ratio on the Morphology and Tensile Properties of ABS Resins. J. Soc. Mater. Sci., Jpn..

[19] Li G., Lu S., Pang J., Bai Y., Zhang L., Guo X. (2012). Preparation,
Microstructure and Properties of ABS Resin with Bimodal Distribution
of Rubber Particles. Mater. Lett..

[20] Merkel M. P., Dimonie V. L., El-Aasser M. S., Vanderhoff J. W. (1987). Morphology
and Grafting Reactions in Core/Shell Latexes. J. Polym. Sci. Part A Polym. Chem..

[21] Sohn S., Kim S., Sung I. H. (1996). Synthesis
and Application of Poly (Butadiene-g-Acrylonitrile-Styrene)
Core-Shell Rubber Particles for Use in Epoxy Resin Toughening. I.
Synthesis of Poly­(Butadiene-g-Acrylonitrile-Styrene). J. Appl. Polym. Sci..

[22] Giaconi G. F., Castellani L., Maestrini C., Ricco T. (1998). Development of Toughness
in ABS Resins. Polymer..

[23] Daniels E. S., Dimonie V. L., El-Aasser M. S., Vanderhoff J. W. (1990). Preparation
of ABS (Acrylonitrile/Butadiene/Styrene) Latexes Using Hydroperoxide
Redox Initiators. J. Appl. Polym. Sci..

[24] Matsuo M., Nozaki C., Jyo Y. (1969). Fine Structures
and Fracture Processes
in Plastic/Rubber Two -phase Polymer Systems. I. Observation of Fine
Structures under the Electron Microscope. Polym.
Eng. Sci..

[25] Hu R., Dimonie V. L., El-Aasser M. S. (1997). Preparation and Characterization
of Poly­(Butadiene-Stat-Styrene)/Poly­(Styrene-Stat-Acrylonitrile) Structured
Latex Particles. J. Appl. Polym. Sci..

[26] Bertin M., Marin G., Montfort J. (1995). Viscoelastic
Properties of Acrylonitrile-Butadiene-Styrene
(ABS) Polymers in the Molten State. Polym. Eng.
Sci..

[27] Hipps H. N., Poehlein G. W., Schork F. J. (2001). Developing a Continuous Emulsion
PBD-Graft- SAN Polymerization Process: Factors Impacting Morphology
Control. Polym. React. Eng..

[28] Aoki Y. (1987). Dynamic Viscoelastic
Properties of ABS Polymers in the Molten State. 5.Effect of Grafting
Degree. Macromolecules..

[29] Aguirre M., Paulis M., Leiza J. R., Guraya T., Iturrondobeitia M., Okariz A., Ibarretxe J. (2013). High-Solids-Content
Hybrid Acrylic/CeO2
Latexes with Encapsulated Morphology Assessed by 3D-TEM. Macromol. Chem. Phys..

[30] Chimenti S., Vega J. M., Aguirre M., Garcia-Lecina E., Diez J. A., Grande H.-J., Paulis M., Leiza J. R. (2017). Effective
Incorporation of ZnO Nanoparticles by Miniemulsion Polymerization
in Waterborne Binders for Steel Corrosion Protection. J. Coatings Technol. Res..

[31] Rajabalinia N., Hamzehlou S., Modin E., Chuvilin A., Leiza J. R., Asua J. M. (2019). Coupling
HAADF-STEM Tomography and Image Reconstruction
for the Precise Characterization of Particle Morphology of Composite
Polymer Latexes. Macromolecules.

[32] Frank, J. Introduction: Principles of Electron Tomography. In Electron Tomography; Springer, 2007; pp 1–15.

[33] Midgley P. A., Weyland M. (2003). 3D Electron Microscopy in the Physical
Sciences: The
Development of Z-Contrast and EFTEM Tomography. Ultramicroscopy..

[34] Weyland M., Midgley P. A. (2004). Electron Tomography. Mater. Today..

[35] Rajabalinia N., Ballard N., Hamzehlou S., Leiza J. R., Asua M. (2021). On-Line Control
of the Particle Morphology of Composite Polymer-Polymer Waterborne
Dispersions. Chem. Eng. J..

[36] Mat Web: Material property data Overview of materials for Styrene Acrylonitrile (SAN). https://www.matweb.com/search/DataSheet.aspx?MatGUID=b19565721c534077911ecf643c7cfc94&ckck=1 (accessed 2023–12–02).

[37] AZO materials Styrene Acrylonitrile - SAN https://www.azom.com/article.aspx?ArticleID=891 (accessed 2023–12–02).

[38] Sibur International Polybutadiene rubber https://sibur-int.com/product/rubber/catalog/item395.php#:~:text=Polybutadiene Polybutadiene synthetic is essentially a,(%40%2B25 °C) (accessed 2023–12–02).

[39] Scientific Polymer Products Inc. Density of polymers https://scipoly.com/density-of-polymers-by-density/ (accessed 2023–12–02).

[40] Stubbs J., Tsavalas J., Carrier R., Sundberg D. (2010). The Structural Evolution
of Composite Latex Particles during Starve-Fed Emulsion Polymerization:
Modeling and Experiments for Kinetically Frozen Morphologies. Macromol. React. Eng..

[41] Hamzehlou S., Leiza J. R., Asua J. M. (2016). A New Approach
for Mathematical Modeling
of the Dynamic Development of Particle Morphology. Chem. Eng. J..

[42] Agirre A., Aguirre M., Leiza J. R. (2022). Characterization
of Grafting Properties
of ABS Latexes: ATR-FTIR vs NMR Spectroscopy. Polymer..

