# Supplementary material for: Unraveling the Particle Morphology of ABS Polymer Latexes by 3D STEM
Source: Macromolecules. 2025 Aug 6;58(16):8591–8. doi: 10.1021/acs.macromol.5c00657 (PMC12392729; doi:10.1021/acs.macromol.5c00657)
Supplement: Supplementary file 1 [file ma5c00657_si_001.pdf]

Supporting Information for:

Unravelling the particle morphology of ABS polymer latexes by 3D STEM

Ainara Agirre[1], Evgeny Modin[2], Andrey Chuvilin[2,3], Miren Aguirre[1], Jose R. Leiza*[1]*

[1] POLYMAT eta Kimika Aplikatua saila, Kimika Fakultatea, University of the Basque Country (UPV/EHU), Joxe Mari Korta Zentroa, Tolosa Hiribidea 72, 20018, Donostia-San Sebastian (Spain)

[2] CIC nanoGUNE BRTA, Tolosa Hiribidea 76, 20018, Donostia-San Sebastian (Spain)

[3] IKERBASQUE, Basque Foundation for Science, Plaza Euskadi 5, 48009 Bilbao, Spain

**Email: jrleiza@ehu.eus ; miren.aguirre@ehu.eus*

Primary and intermediate data of the tomographic analysis

Figure S1 presents snapshots of the tilt's series taken at different angles.

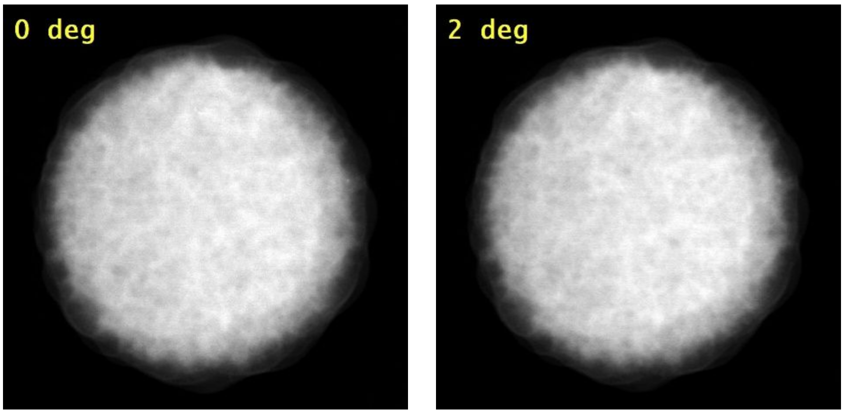

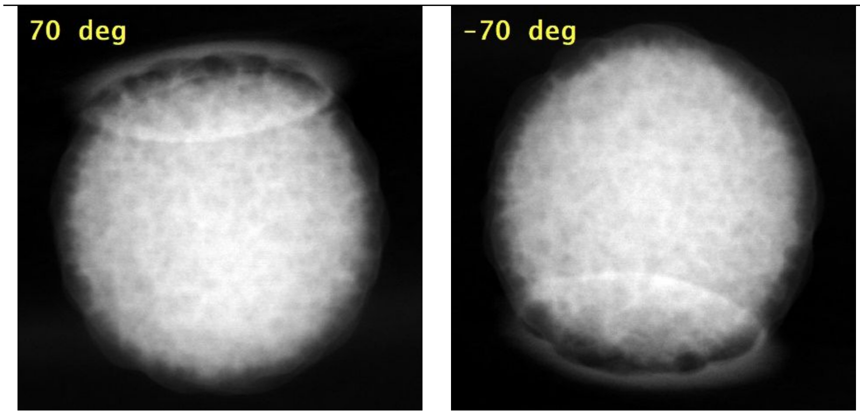

Figure S1. Snapshoots images of the tomographic analysis carried out at relevant angles. A video of the whole raw tilt series obtained by STEM tomography is available at Zenodo (https://doi.org/10.5281/zenodo.15554119)

The reconstructed 3D image video of the analysed particle has been uploaded with the revised manuscript (video.tomo.ser.mp4).

Lower magnification STEM images

Figure S2 displays additional images of ABS particles taken with a HAADF detector. Since the latex was highly diluted to have the particles well separated the number of particles is small. Images with a higher number of particles are also displayed in Figure S3 but using a bright field detector.

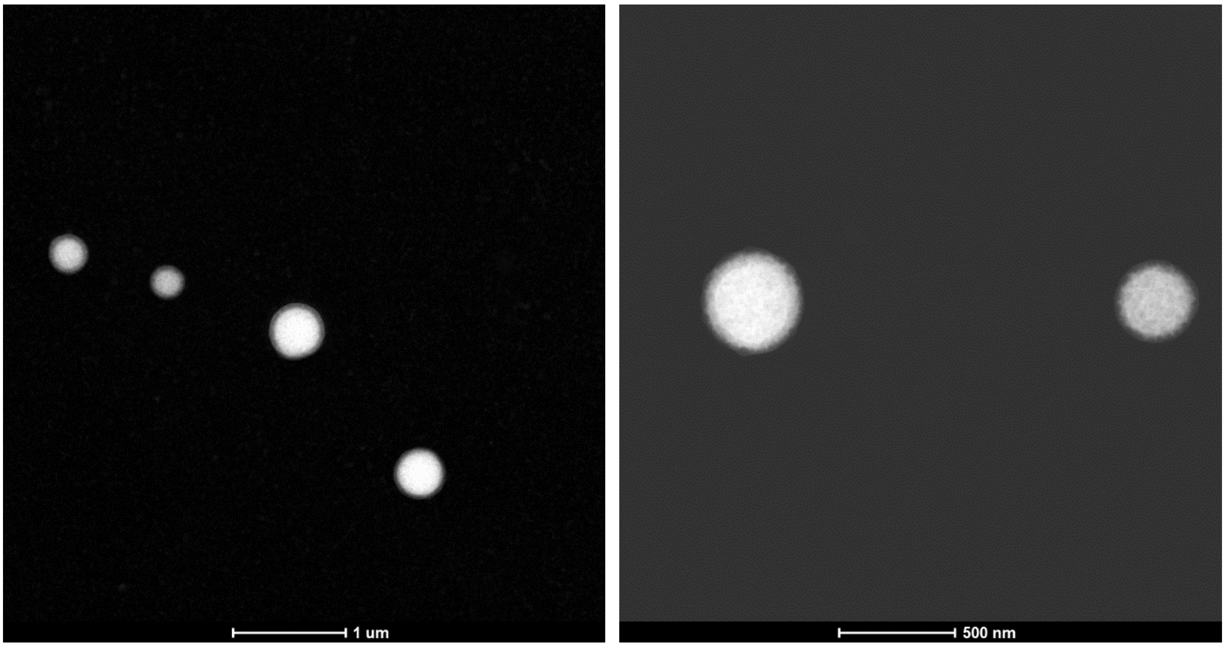

Figure S2. Low magnification images of the ABS latex sample in STEM-HAADF.

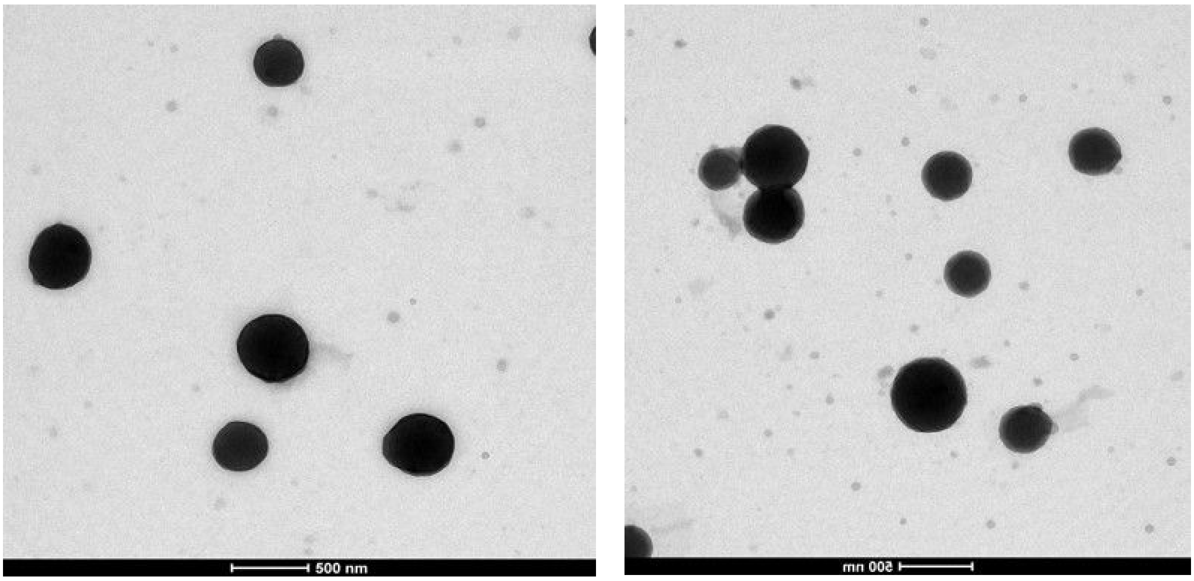

Figure S3. Low magnification images of the ABS latex sample in STEM-HAABF.

Statistical information of the cluster sizes distributions in the particle

Figure S3a is the distribution of the number of clusters as a function of the radial distance and Figure S3b is the same distribution for clusters of different sizes.

a)

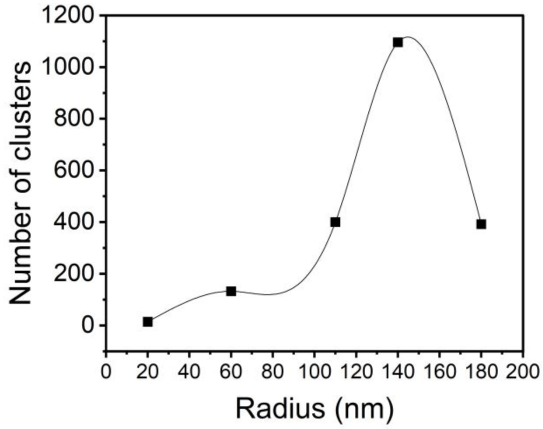

b)

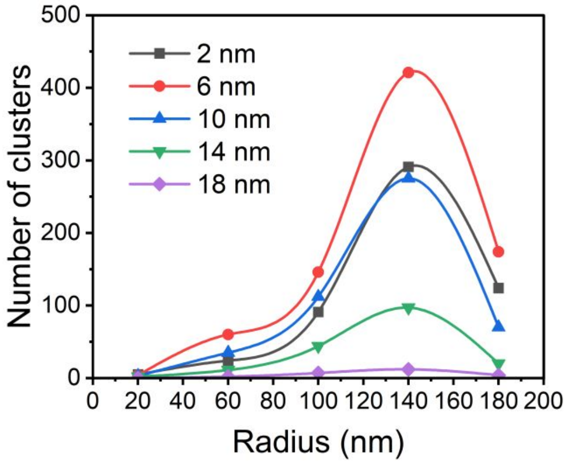

Figure S3. a) Number distribution of clusters as a function of the radial distance and b) Number distribution of clusters of selected sizes as a function of the radial distribution.

The radial distributions clearly indicate that the majority of internal clusters are located between 100 and 180 nm radial distance: namely, towards the surface of the particle. This is further indication that aqueous phase radicals once absorbed in the particle diffuse inward, but they are grafted before they can reach the center of the particle.

SEC and AF4/MALS/RI analysis of the soluble fraction of the ABS latex

The molar mass and molar mass distribution of the soluble fraction of the ABS latex was determined by SEC/RI and AF4/MALS/RI, and the values are shown in Table S1. The SEC set up consisted of a pump (LC-20A, Shimadzu), auto-sampler (Waters 717), a differential refractometer (Waters 2410), three columns

in series (Styragel HR2, HR4 and HR6) with pore sizes ranging from 10^2 to 10^6 Å and a Refractive Index (RI) detector. The chromatograms were obtained at 35 ^oC using THF flow rate of 1 mL/min. The equipment was calibrated using polystyrene standards (5th order universal calibration), so the molar masses were referred to PS. Toluene was used as marker.

The AF4 technique worked on fractionation in combination with a Multi Angle Light Scattering (MALS) and a Refractive Index (RI detector), AF4/MALS/RI. In this work, the separation was carried out on a 27.5 cm trapezoidal channel mounted on PEEK (polyether ether ketone) upper and lower blocks with a stainless-steel frit. The channel thickness spacer was 490 µm. The accumulation wall was a regenerated cellulose membrane with a cut-off molar mass of 10^3 Da. AF4/MALS/RI flow control was maintained with a Wyatt Eclipse 3 AF4/MALS/RI Separation System controller (Wyatt Technology, USA). The MALS detector was a Dawn Heleos II (Wyatt Technology, USA) and the RI detector was an Optilab Rex (Wyatt Technology, USA).

The soluble fraction yield average molar masses in the range of 10^4 – 10^5 Da. This was expected because the soluble fraction is made of ungrafted SAN chains. The molar mass recovered from the AF4/MALS/RI is higher and narrower than that measured by SEC/RI because the AF4 fractionation has a cut-off membrane from which small chains pass and hence are not analysed. Besides, the MALS signal at lower molar masses is weak and hence part of the chromatogram cannot be analysed.

In addition, from the AF4/MALS/RI analysis the radius of gyration was calculated for the soluble chains. As reported in Table S1 a value of 15.7 nm was calculated. Note that since a fraction of small chains is not analysed (they passed through membrane used in the fractionation channel) the value obtained is likely overestimating the overall radius of gyration of the soluble chains.

Table S1. Weight-average molar mass ($\overline{\mathbf{M}}_w$) and dispersity values of the soluble fraction of R1-Ref and R2-Ref ABS latexes measured by GPC and AF4/MALS/RI.

	SEC		AF4/MALS/RI		
	$\overline{M}_w$ (10^4) (Da)	Đ	$\overline{M}_w$ (10^4) (Da)	Đ	Rz (nm)
ABS $_{\text{soluble fraction}}$	2.8	2.8	4.8	1.6	15.7±4.7

z-average radius of the internal clusters of SAN

From the SAN cluster radius distribution presented in Figure 4b in the main text, the z-average radius was calculated using the following equation:

$$r_z = \frac{\sum n_i r_i^5}{\sum n_i r_i^4}$$

A value of $r_z = 10.6\ nm$ is obtained.
